# Deep learning models for predicting the survival of patients with chondrosarcoma based on a surveillance, epidemiology, and end results analysis

**DOI:** 10.3389/fonc.2022.967758

**Published:** 2022-08-22

**Authors:** Lizhao Yan, Nan Gao, Fangxing Ai, Yingsong Zhao, Yu Kang, Jianghai Chen, Yuxiong Weng

**Affiliations:** ^1^ Department of Hand Surgery, Union Hospital, Tongji Medical College, Huazhong University of Science and Technology, Wuhan, China; ^2^ Department of Orthopaedics, Liyuan Hospital, Tongji Medical College, Huazhong University of Science and Technology, Wuhan, China

**Keywords:** chondrosarcoma, survival analysis, machine learning, DeepSurv, deep learning

## Abstract

**Background:**

Accurate prediction of prognosis is critical for therapeutic decisions in chondrosarcoma patients. Several prognostic models have been created utilizing multivariate Cox regression or binary classification-based machine learning approaches to predict the 3- and 5-year survival of patients with chondrosarcoma, but few studies have investigated the results of combining deep learning with time-to-event prediction. Compared with simplifying the prediction as a binary classification problem, modeling the probability of an event as a function of time by combining it with deep learning can provide better accuracy and flexibility.

**Materials and methods:**

Patients with the diagnosis of chondrosarcoma between 2000 and 2018 were extracted from the Surveillance, Epidemiology, and End Results (SEER) registry. Three algorithms—two based on neural networks (DeepSurv, neural multi-task logistic regression [NMTLR]) and one on ensemble learning (random survival forest [RSF])—were selected for training. Meanwhile, a multivariate Cox proportional hazards (CoxPH) model was also constructed for comparison. The dataset was randomly divided into training and testing datasets at a ratio of 7:3. Hyperparameter tuning was conducted through a 1000-repeated random search with 5-fold cross-validation on the training dataset. The model performance was assessed using the concordance index (C-index), Brier score, and Integrated Brier Score (IBS). The accuracy of predicting 1-, 3-, 5- and 10-year survival was evaluated using receiver operating characteristic curves (ROC), calibration curves, and the area under the ROC curves (AUC).

**Results:**

A total of 3145 patients were finally enrolled in our study. The mean age at diagnosis was 52 ± 18 years, 1662 of the 3145 patients were male (53%), and mean survival time was 83 ± 67 months. Two deep learning models outperformed the RSF and classical CoxPH models, with the C-index on test datasets achieving values of 0.832 (DeepSurv) and 0.821 (NMTLR). The DeepSurv model produced better accuracy and calibrated survival estimates in predicting 1-, 3- 5- and 10-year survival (AUC:0.895-0.937). We deployed the DeepSurv model as a web application for use in clinical practice; it can be accessed through https://share.streamlit.io/whuh-ml/chondrosarcoma/Predict/app.py.

**Conclusions:**

Time-to-event prediction models based on deep learning algorithms are successful in predicting chondrosarcoma prognosis, with DeepSurv producing the best discriminative performance and calibration.

## Introduction

Chondrosarcoma accounts for 20-30% of primary bone tumors in adulthood and is the second most frequently occurring bone sarcoma behind osteosarcoma (1). Compared to Ewing sarcoma and osteosarcoma, chondrosarcoma is a less malignant disease, with most patients living for 10 years following standard therapy (2). The clinical presentation of chondrosarcoma varies. 90% are conventional chondrosarcomas and 90% of these are low to intermediate-grade tumors. These tumors are slow growing, less likely to metastasize and relatively insensitive to both chemotherapy and radiotherapy (3). The remaining 10-8% of non-conventional tumors are further classified into five subtypes: myxoid, mesenchymal, dedifferentiated, juxtacortical, and clear cell. Those sarcomas (including 5-10% of high-grade conventional chondrosarcomas) can be highly malignant and aggressive, with a higher probability of metastasis, leading to poorer outcomes for patients (4).

Several prognostic models have been created utilizing multivariate Cox regression or machine-learning approaches to predict the 3- and 5-year survival of patients with chondrosarcoma (5–8). Among these models, the nomogram is a frequently used method for integrating and measuring different significant clinical variables of patients when assessing the odds of occurrence of events using the Cox proportional hazards (CoxPH) model. However, one of the underlying assumptions regarding the CoxPH model is that each predictor variable has the same effect at each follow-up time point; however, this overlooks changes in the effect of predictor factors on individual patients at different time points. Additionally, these models use linearity assumptions rather than conducting nonlinear analyses that represent clinical aspects in the real world. As a result, improved solutions focusing on nonlinear variables are required. The Skeletal Oncology Research Group (SORG) algorithm was proposed (5), which trained several binary classification-based machine learning models using the National Cancer Institute’s Surveillance, Epidemiology, and End Results (SEER) data to predict 5-year survival, with the highest AUC being 0.868. The algorithm was subsequently validated on data from two external datasets (9, 10) and showed good performance. Although the SORG algorithm achieves better prediction performance than traditional methods by assessing the nonlinear relationships between variables, its limitations are also obvious. Firstly, it applied a machine learning method to survival data by simplifying the prediction as a binary classification problem; this approach lacks the interpretability and flexibility provided by modeling the probabilities of events as a function of time (11). Secondly, it was trained using data from the SEER database between 2004 and 2010, but data from 2011 to 2018 are already available in the SEER database. Since treatment strategies have evolved in recent years, the patient’s clinical characteristics may have changed. Thirdly, the surgical treatment of patients (one of its input features) is not classified in detail. However, the type of surgery may be associated with survival rates (5).

In order to address all of the above-mentioned issues concerning survival predictions, new approaches for combining machine learning methods with survival models have been proposed. Katzman et al. (12) integrated the Cox proportional hazards model with neural networks (DeepSurv) and showed that this novel approach was able to outperform classical Cox models (13, 14). The DeepSurv model used the negative log partial likelihood function to assess patients’ survival hazards, utilizing a core hierarchical structure composed of fully connected feed-forward neural networks with a single output node. Yu et al. (15) proposed the Linear Multi-Task Logistic Regression (MTLR) model—an extension of binomial log-likelihood—for jointly modeling a series of binary labels representing event indicators. It is a collection of logistic regression models constructed at several different time intervals that can be used to assess the probability that the event of interest occurred within each interval. The neural MTLR (N-MTLR) (16) model is based on the MTLR technique but utilizes a deep learning architecture that considers nonlinear relationships in datasets; this method has been shown to outperform the MTLR model in the majority of cases (16). The random survival forest (RSF) model is an extension of the random forest model that takes censoring into account and has been used as a benchmark for method comparison in many pieces of literature (11).

This study aimed to develop models for predicting the overall survival (OS) of patients with chondrosarcoma using the Cox proportional hazards model and three machine learning algorithms and compared the predictive performance of these methods. In addition, the best algorithm will be deployed as an accessible web-based app for clinical use.

## Methods

### Patient population and data collection

**Figure 1:**
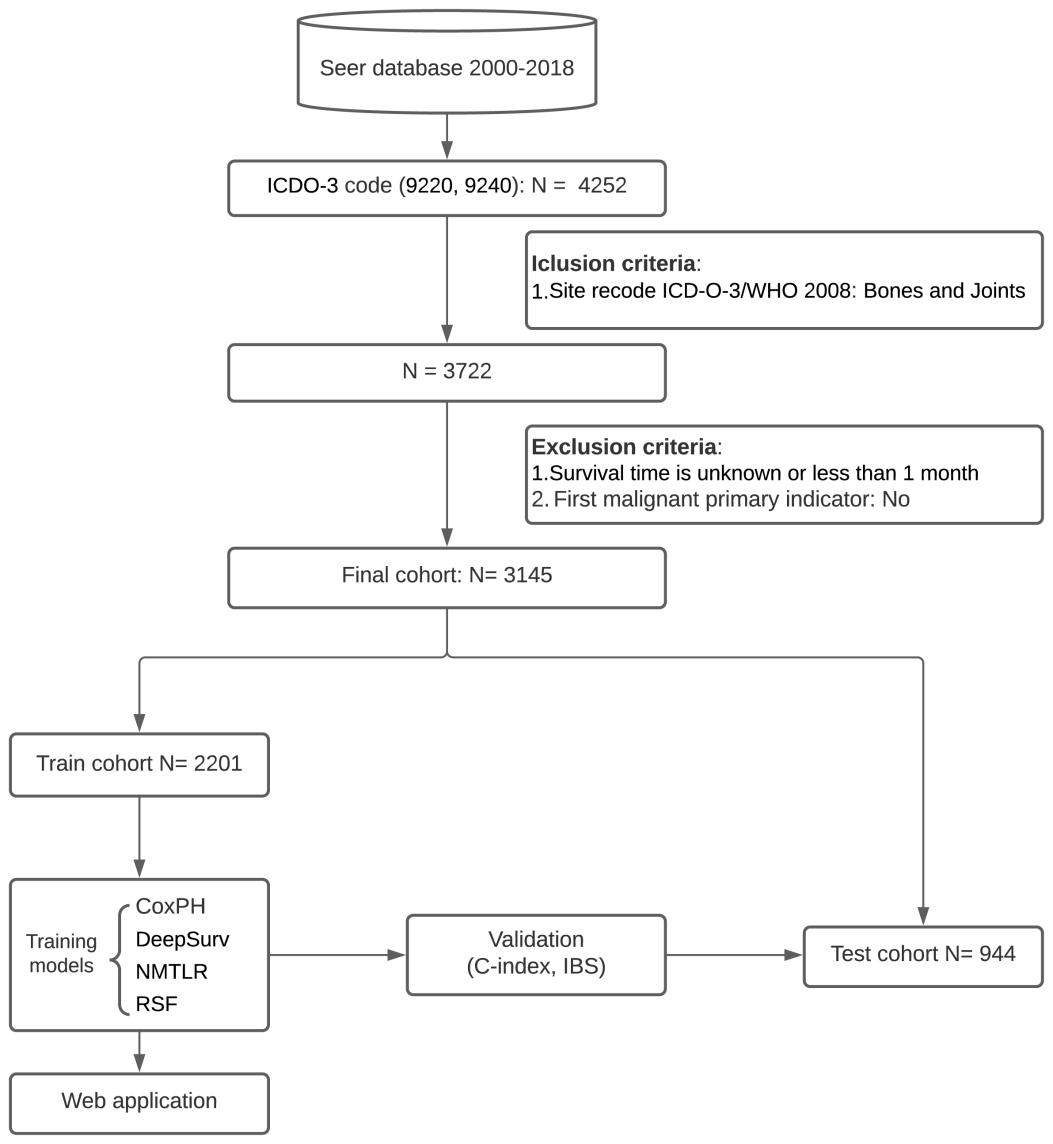
Study profile and analysis pipeline.

Patients were identified from the SEER database for the period 2000-2018 for this retrospective cohort study. The SEER database collects information from 18 cancer registries and covers approximately 28% of the total US population. SEER*Stat software (Version 8.4.0; National Cancer Institute, Bethesda, MD) was used to extract information from the SEER database. We collected the baseline information of cases (year of diagnosis, gender, age), tumor characteristics (size, number, histologic type, grade, primary site, tumor extension, distant metastasis site, and stage) and treatment details (surgical type, radiotherapy and chemotherapy). The inclusion criteria were as follows: (1) patients have a confirmed diagnosis of chondrosarcoma according to the third edition of the International Classification of Diseases for Oncology (ICD-O-3), morphological code (9220, 9240); (2) bones and joints are the primary site (site recode ICD-O-3/WHO 2008 = Bones and Joints). The exclusion criteria were as follows: (1) survival time is unknown or less than one month; (2) chondrosarcoma was not identified as the primary tumor (first malignant primary indicator = No). A flowchart of the detailed selection process is presented in **Figure 1**.

### Variable’s definitions

The following variables are extracted from the SEER database: Year of diagnosis, Age, Gender, Histological type, Primary site, Stage, Grade, Surgery, Radiotherapy, Chemotherapy, Tumor size, Number of tumors, Tumor extension, Distant metastasis, Survival months, Status. The original name of variables in the SEER database and the specific details of each categorical variable was shown in **Supplementary Material E1**, section S1. Until 2018, The grading system in SEER has been consistent throughout all the years of data collection and consists of a four‐tier system with grade IV corresponding to undifferentiated tumors in addition to the common grades I (well), II (moderate) and III (poorly). The new grading strategy “Grade Clinical (2018+)” has been implemented in the SEER database since 2018, which consists of three grades and explicitly mentions that Grade 3 includes undifferentiated tumors.

### Deep learning model design

The source code of model development is available on GitHub (https://github.com/WHUH-ML/Chondrosarcoma).

### Feature selection

Collinearity occurs when two features have a strong association with one another. Highly correlated features should be avoided since they increase computational cost and effort and they overfit the model. Thus, the cor function in the stats R package was used to calculate correlations between features, with a Pearson’s correlation value of 0.7 indicating that features are highly collinear. In addition, univariate and multivariate Cox regression were used to assess the potential features.

### Data preprocessing

Binary categorical features were coded as 0 and 1. Ordinal features were encoded as ordinal numeric values, and categorical features were one-hot encoded. We implemented the nonparametric missForest imputation method for handling missing data, which imputes missing values based on random forest predictions. Continuous features were standardized using the StandardScaler function from the sklearn preprocessing library.

### Model development

The primary predicted outcome was overall survival (OS). Three algorithms—two based on neural networks (DeepSurv, NMLTR) and one on ensemble learning (RSF)—were selected for training. Meanwhile, a multivariate CoxPH model was also constructed for comparison. The dataset was randomly divided into training and testing datasets at a ratio of 7:3.

### Hyperparameter tuning

It was essential to find the best configuration for our proposed network, including network architecture and hyperparameter values. Hyperparameter tuning was conducted through a 1000-repeated random search with 5-fold cross-validation on the training dataset. The concordance index (C-index) was used to evaluate the performance of models with different combinations of hyperparameters.

### Model evaluation

The accuracy of models was determined using C-index, which is a correlation coefficient between predicted survival risks and observed survival times. A C-index value of 0.5 indicates that the prediction is random, whereas a C-index value of 1.0 indicates excellent prediction. The difference between the two models’ C-index was tested using Kang’s method (17). Brier scores were also obtained; they indicate the mean square difference between observed patient status and predicted survival probability and are always between 0 and 1, with 0 being the best possible result. A model with a Brier score of less than 0.25 is considered useful in practice. The Integrated Brier Score (IBS) was also calculated to determine the models’ overall performance across all available periods. The 1-, 3-, 5- and 10-year OS were calibrated using a calibration curve, comparing expected and observed survival. In order to assess the time-dependent sensitivities and specificities of the models, receiver operating characteristic (ROC) curves were generated, and the area under the curve (AUC) values were calculated for 1-, 3-, 5- and 10-year survival.

### Feature importance

To determine the association between individual features and model performance, we estimated the importance of each feature within the test set by replacing the feature data with random numbers (18). The performance of the models, as measured by the concordance index, was then computed using the data after replacement to assess the importance of each feature.

### Model deployment

The algorithm with the best performance was deployed using the Streamlit package in Python to create an interactive web-based tool for practical use.

### Statistical analysis

All continuous variables in clinical data are displayed as the mean value ± standard deviation (SD). Frequencies and percentages are used to characterize categorical variables. The chi-square test and unpaired two-side t-test were utilized to examine the differences in variables across groups. The R programming language (version 4.1.2) was used to carry out data preprocessing and plotting. The machine learning models were constructed using the PySurvival package in the Python programming language (version 3.6.8).

## Results

### Basic characteristics

A total of 3145 chondrosarcoma patients registered in the SEER database from 2004 to 2015 were finally enrolled in this study. The patient demographic characteristics are shown in **Table 1**. 1483 cases were female (47%), and 1662 were male (53%); the mean age was 52 ± 18 years. In terms of the primary site of tumors, 1595 of them were in the extremities (51%), 702 in the axial skeleton (22%), and 848 in other joints and bones (27%). 1033 cases were well-differentiated (39%), 1099 were moderately differentiated (41%), 319 were poorly differentiated (12%), and 208 were undifferentiated (7.8%). 393 cases did not undergo surgery (13%), 1066 underwent a local treatment (35%), 1243 underwent a radical excision with limb salvage (41%), and 358 underwent amputation surgery (12%). The mean overall survival (OS) was 83 ± 67 months, and 904 patients died (29%).

**Table 1:** Patient demographic, disease, treatment characteristics, and Cox regression analysis.

	Overall	Univariate Cox			Multivariate Cox		
Characteristic	N = 3,145^1^	HR^2^	95% CI^2^	P-value	HR^2^	95% CI^2^	P-value
**Year of diagnosis**				0.23			0.17
* 2004-2010*	1,768 (56%)	—	—		—	—	
* 2011-2015*	1,377 (44%)	1.10	0.94, 1.27		0.85	0.68, 1.07	
**Age**	52 (18)	1.05	1.05, 1.06	**<0.001**	1.04	1.03, 1.05	**<0.001**
**Gender**				**<0.001**			**<0.001**
* Female*	1,483 (47%)	—	—		—	—	
* Male*	1,662 (53%)	1.48	1.29, 1.69		1.58	1.27, 1.96	
**Histological type**				**<0.001**			**<0.001**
* Conventional*	2,879 (92%)	—	—		—	—	
* Dedifferentiated*	266 (8.5%)	6.30	5.34, 7.42		1.96	1.42, 2.69	
**Primary site**				**<0.001**			**0.018**
* Extremity*	1,595 (51%)	—	—		—	—	
* Axial skeleton*	702 (22%)	1.60	1.37, 1.86		1.09	0.84, 1.42	
* Other*	848 (27%)	0.77	0.65, 0.91		0.72	0.54, 0.95	
**Stage**				**<0.001**			0.80
* I*	1,083 (73%)	—	—		—	—	
* II*	249 (17%)	3.36	2.68, 4.22		1.21	0.69, 2.14	
* III*	15 (1.0%)	1.33	0.49, 3.57		0.73	0.21, 2.49	
* IV*	140 (9.4%)	12.8	10.2, 16.2		1.33	0.46, 3.83	
* Missing*	1,658						
**Grade**				**<0.001**			**0.007**
* Well differentiated*	1,033 (39%)	—	—		—	—	
* Moderately differentiated*	1,099 (41%)	1.75	1.45, 2.11		1.40	1.05, 1.88	
* Poorly differentiated*	319 (12%)	4.18	3.36, 5.22		1.73	0.94, 3.20	
* Undifferentiated*	208 (7.8%)	10.4	8.31, 13.0		2.63	1.38, 5.03	
* Missing*	486						
**Surgery**				**<0.001**			**0.002**
* No*	393 (13%)	—	—		—	—	
* Local treatment*	1,066 (35%)	0.24	0.20, 0.29		0.54	0.37, 0.80	
* Radical excision with limb salvage*	1,243 (41%)	0.33	0.28, 0.39		0.48	0.33, 0.68	
* Amputation*	358 (12%)	0.65	0.53, 0.80		0.62	0.42, 0.90	
* Missing*	85						
**Radiotherapy**				**<0.001**			0.39
* No*	2,822 (90%)	—	—		—	—	
* Yes*	323 (10%)	1.42	1.17, 1.72		1.15	0.84, 1.56	
**Chemotherapy**				**<0.001**			0.18
* No*	2,905 (92%)	—	—		—	—	
* Yes*	240 (7.6%)	4.92	4.14, 5.83		1.26	0.90, 1.75	
**Tumor size, mm**	81 (60)	1.00	1.00, 1.01	**<0.001**	1.00	1.00, 1.00	**<0.001**
* Missing*	1,552						
**Number of tumors**				0.28			0.23
* 1*	2,867 (91%)	—	—		—	—	
* > 1*	278 (8.8%)	1.12	0.91, 1.37		0.82	0.59, 1.14	
**Tumor extension**				**<0.001**			**0.002**
* No break in periosteum*	553 (29%)	—	—		—	—	
* Extension beyond periosteum*	1,251 (67%)	2.27	1.81, 2.85		1.50	1.12, 2.00	
* Further extension*	75 (4.0%)	4.73	3.28, 6.82		2.30	1.41, 3.75	
* Missing*	1,266						
**Distant metastasis**				**<0.001**			**0.012**
* No*	1,792 (93%)	—	—		—	—	
* Yes*	128 (6.7%)	9.98	8.07, 12.4		3.15	1.11, 8.93	
* Missing*	1,225						
**Survival months**	83 (67)						
**Status**								
* Alive*	2,241 (71%)						
* Dead*	904 (29%)						

^1^n (%); Mean (SD).

^2^HR = Hazard Ratio, CI = Confidence Interval. P values are bolded to indicate they are less than 0.05.

### Feature selection and data preprocessing

**Figure 2:**
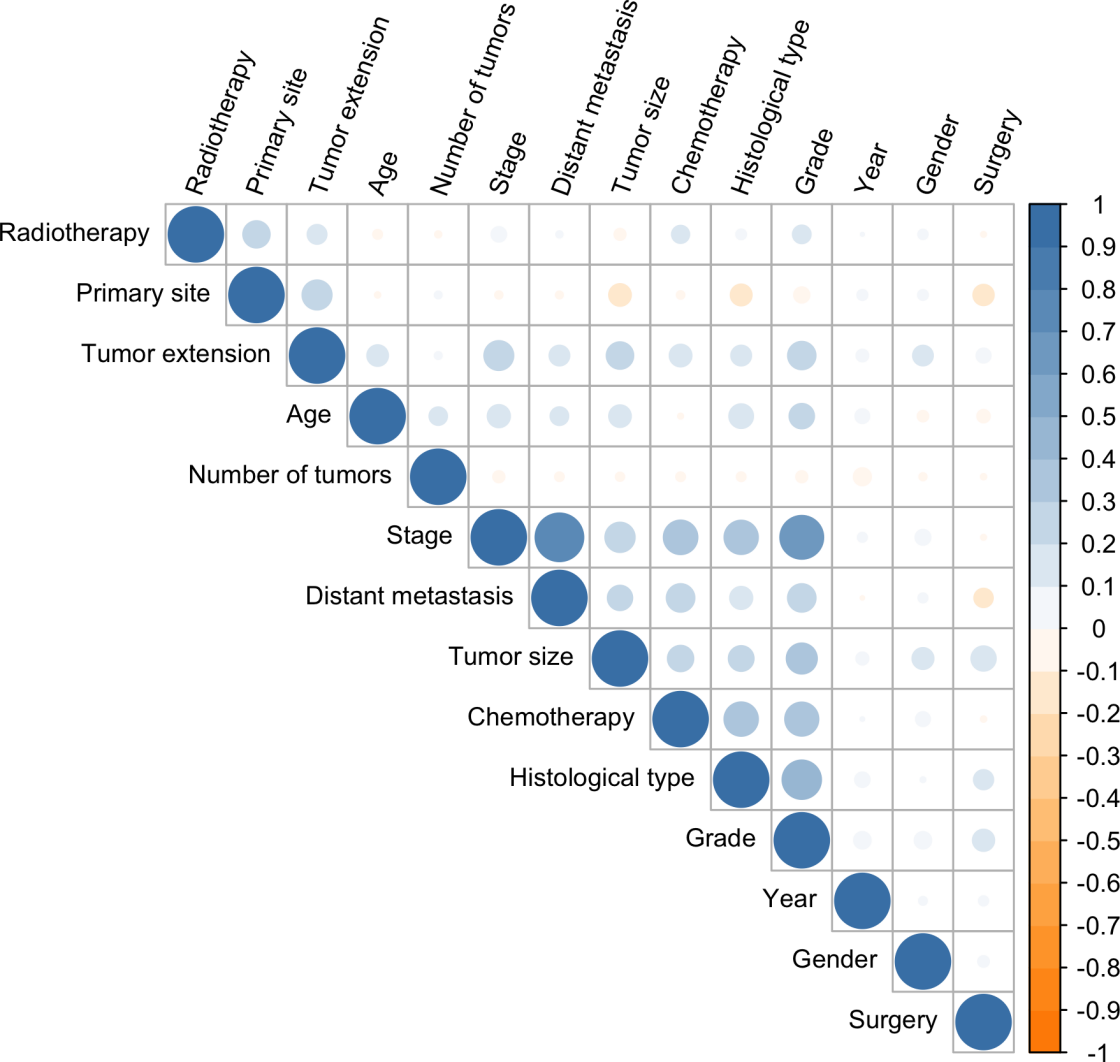
Correlation coefficients for each pair of variables in the data set. The estimated correlation values are distributed within the range of -1 to +1. They are represented by color depth, with a number closer to either end value implying a stronger negative correlation or positive correlation.

In the univariate Cox regression, OS was significantly associated with most features except for the year of diagnosis and the number of tumors (**Table 1**). For the multivariate Cox regression, age, gender, histological type, primary site, grade, surgery, tumor size, tumor extension, and distant metastasis were independent factors for OS (P<0.05). Results of the collinearity analysis showed high collinearity between stage and distant metastasis, and between stage and grade (**Figure 2**). Considered together, we ultimately included nine features (age, gender, histological type, primary site, grade, surgery, tumor size, tumor extension and distant metastasis) in the model development. The dataset was divided into two subsets—training set and testing set; 2203 cases were used for the training set, and the remaining 942 cases were used for the test set (**Table 2**).

**Table 2:** Characteristic distribution of data in training sets and test sets.

	Level	Overall	Train	Test	P-value
**Total**		3145	2203	942	
**Age (mean (SD))**		51.58 (17.53)	51.70 (17.41)	51.29 (17.82)	0.547
**Gender (%)**	Female	1483 (47.2)	1036 (47.0)	447 (47.5)	0.857
	Male	1662 (52.8)	1167 (53.0)	495 (52.5)	
**Histological type (%)**	Conventional	2879 (91.5)	2025 (91.9)	854 (90.7)	0.274
	Dedifferentiated	266 (8.5)	178 (8.1)	88 (9.3)	
**Primary site (%)**	Extremity	1595 (50.7)	1121 (50.9)	474 (50.3)	0.395
	Axial skeleton	702 (22.3)	502 (22.8)	200 (21.2)	
	Other	848 (27.0)	580 (26.3)	268 (28.5)	
**Grade (%)**	Well differentiated	1033 (38.8)	725 (38.6)	308 (39.5)	0.933
	Moderately differentiated	1099 (41.3)	782 (41.6)	317 (40.7)	
	Poorly differentiated	319 (12.0)	228 (12.1)	91 (11.7)	
	Undifferentiated	208 (7.8)	145 (7.7)	63 (8.1)	
**Surgery (%)**	None	393 (12.8)	266 (12.4)	127 (14.0)	0.571
	Local treatment	1066 (34.8)	762 (35.4)	304 (33.5)	
	Radical excision with limb salvage	1243 (40.6)	874 (40.6)	369 (40.7)	
	Amputation	358 (11.7)	251 (11.7)	107 (11.8)	
**Tumor size, mm (mean (SD))**		80.65 (60.19)	80.96 (62.00)	79.88 (55.47)	0.746
**Tumor extension (%)**	No break in periosteum	553 (29.4)	389 (28.9)	164 (30.8)	0.425
	Extension beyond periosteum	1251 (66.6)	900 (66.8)	351 (66.0)	
	Further extension	75 (4.0)	58 (4.3)	17 (3.2)	
**Distant metastasis (%)**	Not	1792 (93.3)	1280 (93.5)	512 (92.9)	0.721
	Yes	128 (6.7)	89 (6.5)	39 (7.1)	
**Survival months (mean (SD))**		83.16 (66.93)	84.50 (66.86)	80.04 (67.01)	0.087
**Status (%)**	Alive	2241 (71.3)	1572 (71.4)	669 (71.0)	0.882
	Dead	904 (28.7)	631 (28.6)	273 (29.0)	

### Hyperparameter tuning

**Figure 3:**
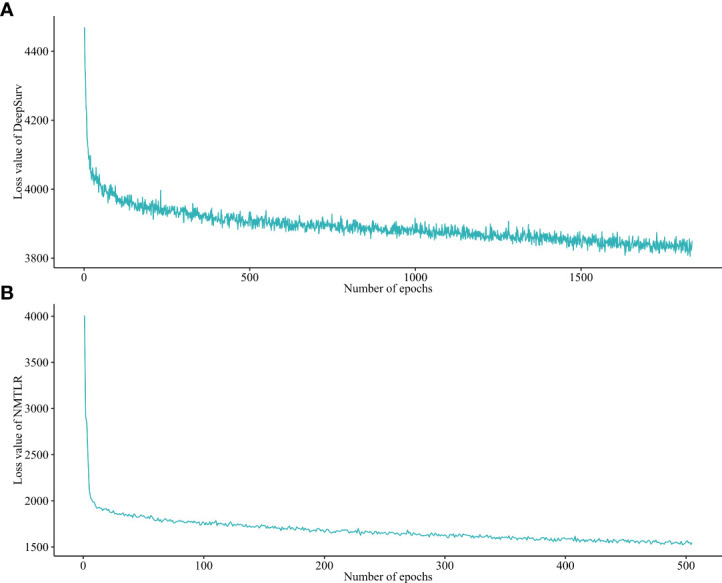
Loss convergence graph for **(A)** DeepSurv, **(B)** neural network multitask logistic regression (N-MLTR) models.

After a 1000-repeated random search with 5-fold cross-validation on the training dataset, we selected those parameters showing the highest average C-index in cross-validation as the optimal parameters. The graph of the loss function for the two neural network models (DeepSurv, and NMTLR) is shown in **Figure 3**. The search space and optimal parameter combinations for models’ hyperparameters are displayed in our open-source code on GitHub (https://github.com/WHUH-ML/Chondrosarcoma).

### Model comparisons

**Figure 4:**
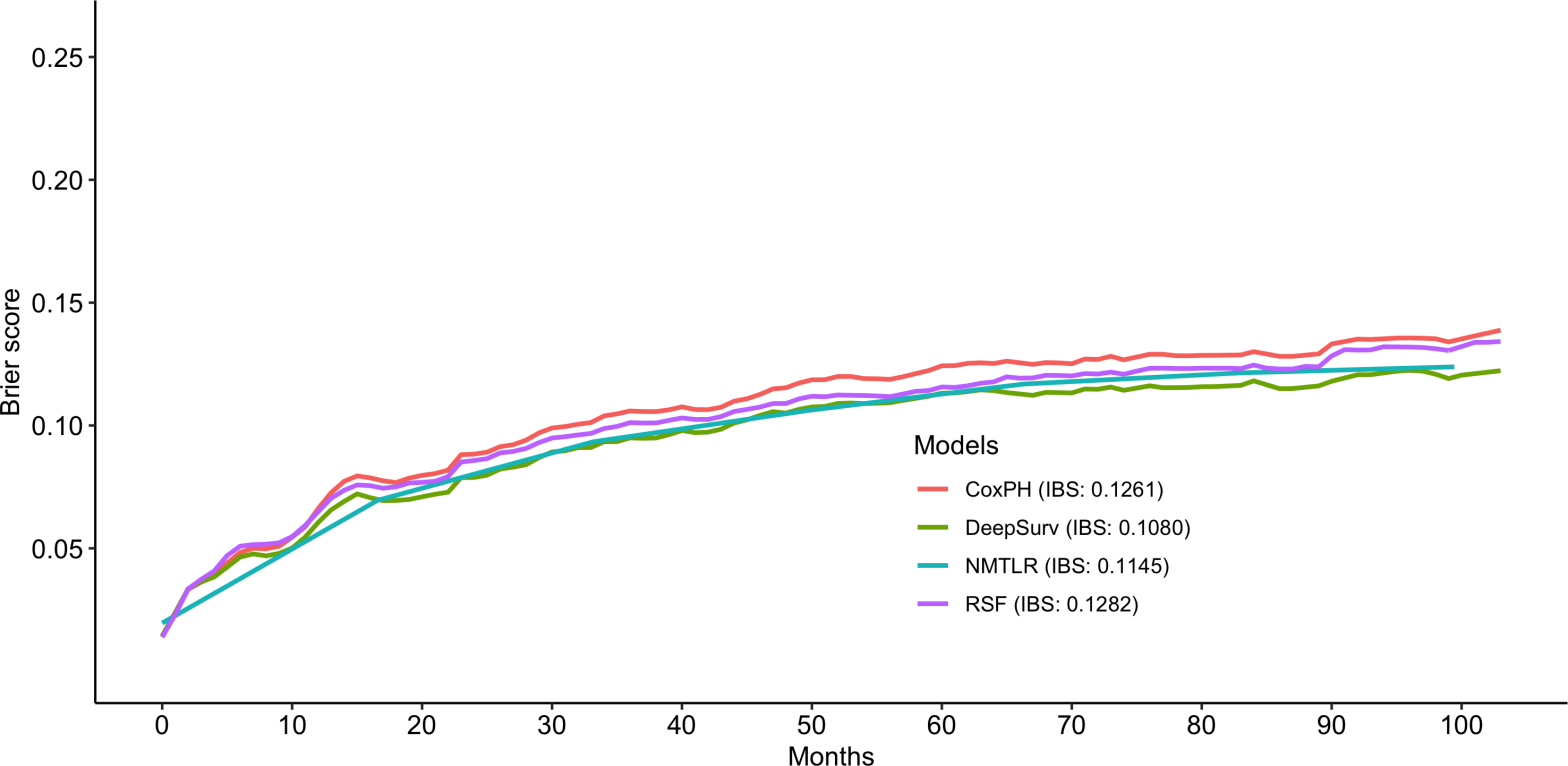
Prediction error curve. As a benchmark, a useful model will have a Brier score below 0.25.

The predictive performance of the machine learning and CoxPH models is shown in **Table 3**. In the test dataset, the three machine learning models showed significant (P < 0.01) better discrimination (C-index of DeepSurv: 0.832; NMLTR: 0.821; RSF: 0.803) compared with the standard CoxPH model (C-index: 0.773); of the three, DeepSurv had the highest C-index of 0.832. The IBS of the four models were 0.108 (DeepSurv), 0.115 (NMLTR), 0.128 (RSF) and 0.126 (CoxPH) (**Figure 4**). There is little difference between the C-index obtained from the training data set (DeepSurv: 0.854; NMLTR: 0.850; RSF: 0.829; CoxPH: 0.782) and that from the test set, indicating that the models do not suffer from overfitting.

**Table 3:** Performance of four survival models.

	C index^a^						
Models	Train^b^	Test^b^	IBS^a^	1-year AUC^a^	3-year AUC	5-year AUC	10-year AUC
CoxPH^a^	0.782	0.773	0.126	0.923 (0.897-0.948)	0.879 (0.852-0.906)	0.865 (0.836-0.893)	0.870 (0.841-0.899)
DeepSurv^a^	**0.854**	**0.832**	**0.108**	**0.937** (0.911-0.962)	**0.907** (0.883-0.931)	**0.895** (0.870-0.920)	**0.896** (0.870-0.921)
NMTLR^a^	0.850	0.821	0.115	0.928 (0.900-0.956)	0.896 (0.870-0.922)	0.889 (0.862-0.915)	0.890 (0.863-0.917)
RSF^a^	0.829	0.803	0.128	0.931 (0.905-0.958)	0.900 (0.873-0.926)	0.889 (0.862-0.916)	0.885 (0.857-0.913)

a CoxPH, standard cox proportional hazards; NMLTR, neural multi-task logistic regression; RSF, random survival forest; IBS, Integrated Brier Score; AUC, area under receiver operating characteristic curve. C index, concordance index.

b C index in train and test dataset are calculated separately, other metrics are calculated only in the test set. Bolded metrics indicate that the metric is the best of the fourgroups.

**Figure 5:**
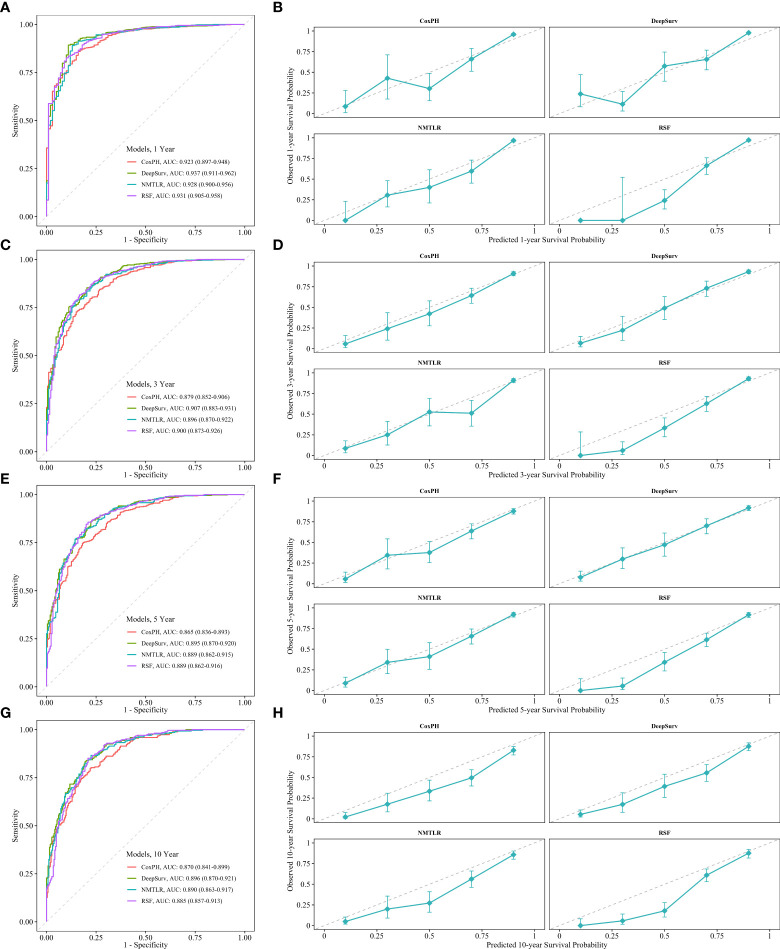
The receiver operating curves (ROC) and calibration curves for 1-, 3-, 5-, 10-year survival predictions. ROC curves for **(A)** 1-, **(C)** 3-, **(E)** 5-, **(G)** 10-year survival predictions. calibration curves for **(B)** 1-, **(D)** 3-, **(F)** 5-, **(H)** 10- year survival predictions.

The calibration plots showed that the consistency between the model’s prediction and the actual observation in terms of the 1-, 3-, 5- and 10-year overall survival rates were best for the DeepSurv model, followed by the NMTLR, CoxPH, and RSF models (**Figure 5**). The AUC was larger for the DeepSurv model than for the three other models (1-year-AUC of DeepSurv: 0.937, NMLTR: 0.896, RSF: 0.900, CoxPH: 0.879; 3-year-AUC of DeepSurv: 0.907, NMLTR: 0.896, RSF: 0.900, CoxPH: 0.879; 5-year-AUC of DeepSurv: 0.895, NMLTR: 0.889, RSF: 0.889, CoxPH: 0.865; 10-year-AUC of DeepSurv: 0.896, NMLTR: 0.890, RSF: 0.885, CoxPH: 0.870) (**Figure 5**). The results showed that the deep learning models—especially the DeepSurv model—were more accurate in predicting the survival prognosis of chondrosarcoma patients than the RSF and classical CoxPH models.

### Feature importance

**Figure 6:**
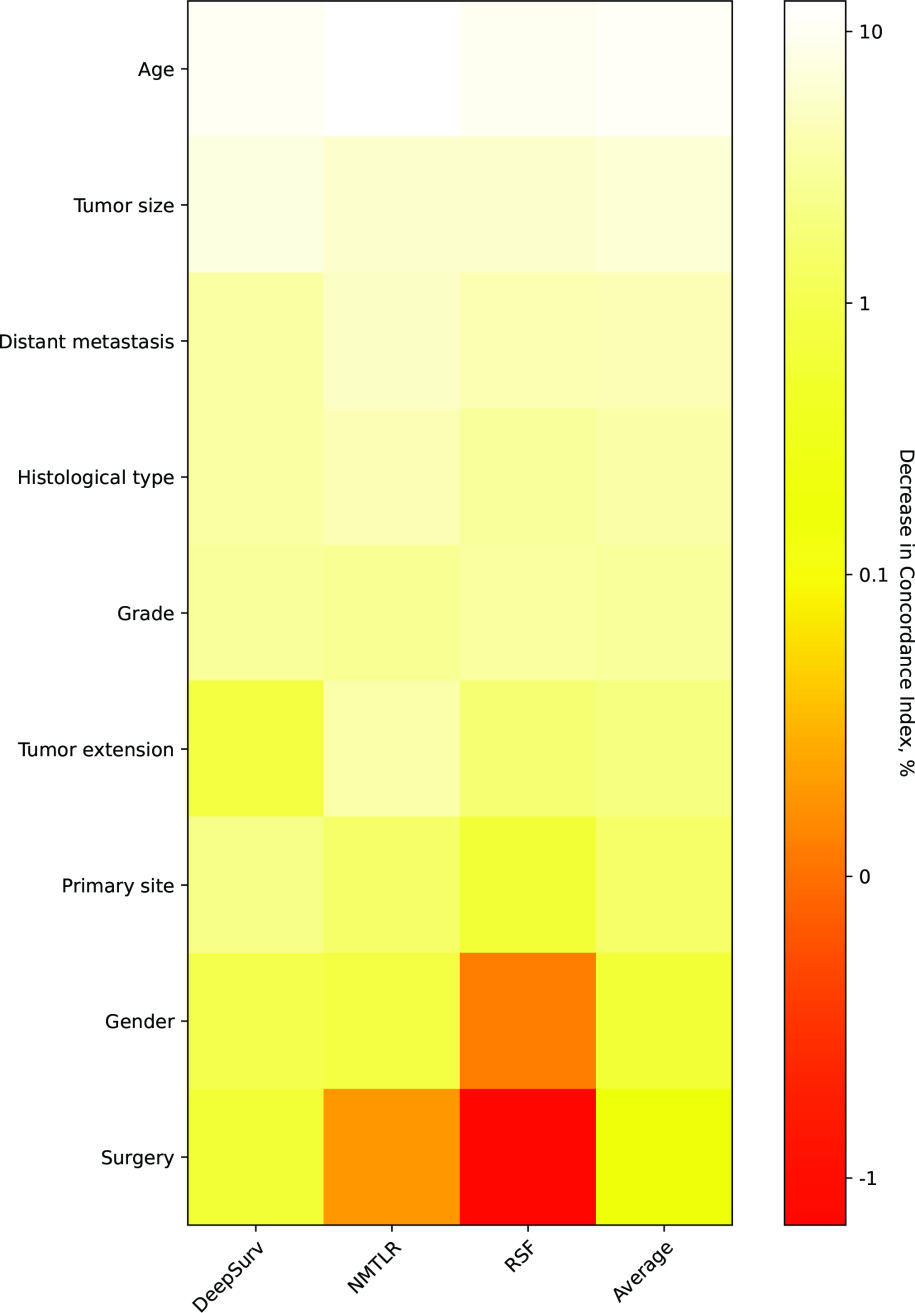
Heatmap of feature importance for DeepSurv, neural network multitask logistic regression (N-MLTR) and random survival forest (RSF) models. The values are expressed as a percentage reduction in the C-index after the value of a feature has been replaced by random numbers. Higher values suggest that a feature is more important in influencing the predictive accuracy of the corresponding deep learning model.

The assessment of feature importance (**Figure 6**) identified features important to model accuracy for prognosis, with a more than 1% mean reduction in the concordance index with replacement data of age, tumor size, distant metastasis, histological type, grade, tumor extension and primary site.

### Algorithm deployment

**Figure 7:**
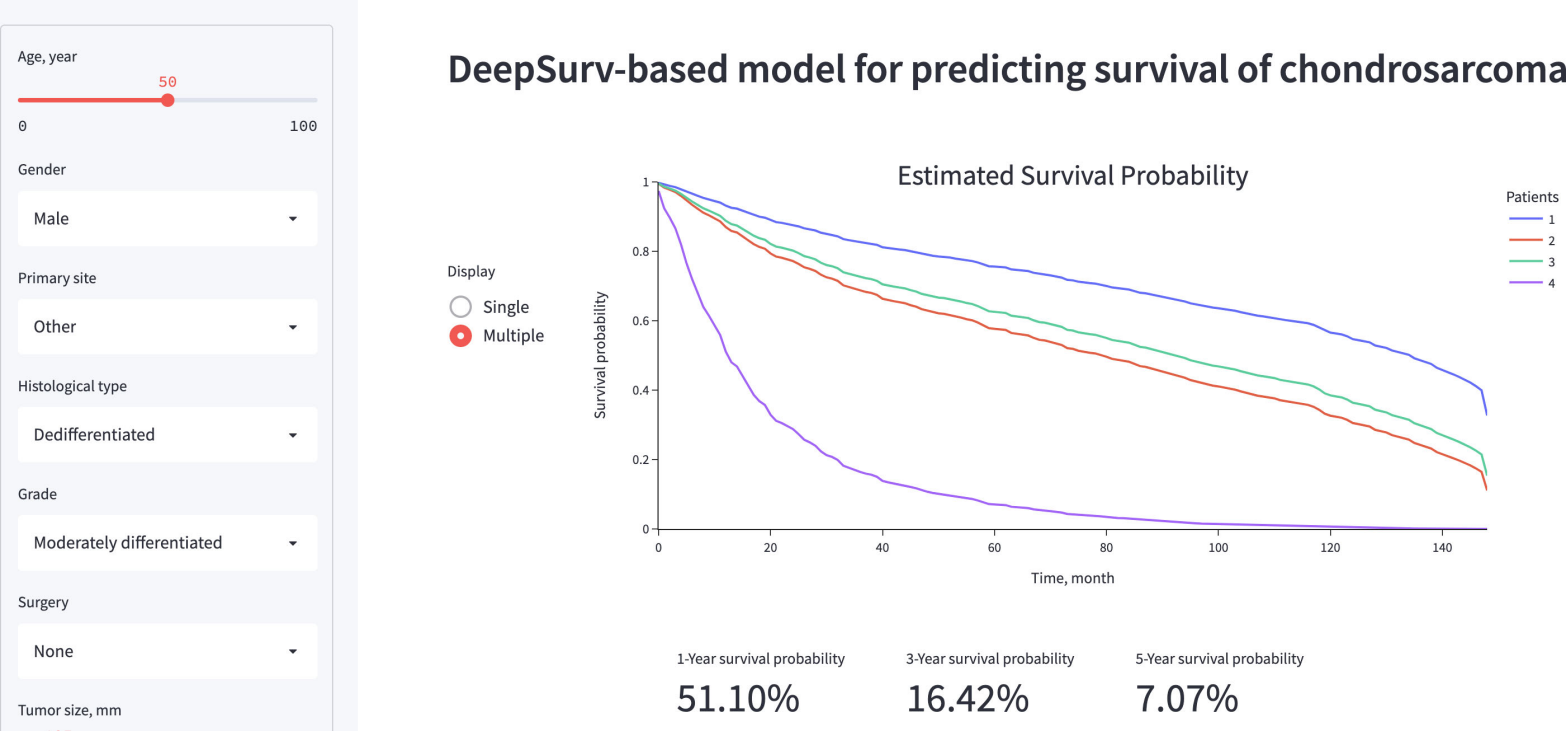
A screenshot of the online web-based application of DeepSurv model.

A visual representation of the functionality and output of the application is presented in **Figure 7**. The web application, which is primarily for research or informational purposes, can be publicly accessed at https://share.streamlit.io/whuh-ml/chondrosarcoma/Predict/app.py.

## Discussion

Accurate prediction for chondrosarcoma survival is crucial for the counseling, follow-up, and treatment planning of patients. Previous studies have revealed various prognostic factors influencing the survival times of patients with chondrosarcoma, including patient age, tumor size, histological type, tumor grade, and metastasis (6, 19–21).. At the same time, increasing amounts of imaging (22, 23) and genetic data (2, 24) are being mined for survival analysis of chondrosarcoma patients. In the face of high-dimensional data, the limitations of the linear relationship between variables assumed by the classical CoxPH model are evident (11). Deep learning is applied to survival analysis due to its ability to comprehensively reveal potential nonlinear relationships in data. In recent years, this method has been gradually improved and successfully applied to clinical (25–27), imaging (28, 29), and genetic data (27). As far as we know, this approach has not been applied to bone tumors. Therefore, we constructed two deep learning models to predict the OS of chondrosarcoma patients and compared the models’ performance with two classical models.

By gathering potentially significant characteristics from the SEER database, this study constructed different models for predicting the survival rates of chondrosarcoma patients. We firstly used Cox proportional hazards regression to identify variables related to the prognosis of 3145 individuals with chondrosarcoma. Age, gender, histological type, original location, tumor grade, surgery, tumor size, tumor extension, and distant metastasis were selected to incorporate in the modeling (p<0.05) (**Table 1**). The two-layer neural network DeepSurv model performed the best, followed by NMTLR, RSF and CoxPH. The C-index values for the DeepSurv model were 0.854 for the training dataset and 0.832 for the test dataset. Roc curves and calibration curves further validated DeepSurv’s performance in terms of discrimination and calibration for predicting 1 -, 3 -, 5 - and 10-year survival. By combining deep learning methods to model the probabilities of events as a function of time, the DeepSurv model outperforms other models when dealing with large samples, multiple variables, and nonlinearity. The best-performing DeepSurv model was incorporated into a user-friendly web-based application that can be accessed for free at https://share.streamlit.io/whuh-ml/chondrosarcoma/Predict/app.py.

Compared to previous studies predicting chondrosarcoma survival, our study showed advantages in terms of discrimination and flexibility. Song (6) used a nomogram to fit data from chondrosarcoma patients in the SEER database prior to 2011 to predict OS, with a c-index of 0.753 for the validation set. In our study, the discrimination of the CoxPH model was slightly improved (0.773), which may be related to the fact that we included more cases and a more detailed classification of surgical procedures. The SORG algorithm proposed by Thio (10) made progress under the task of predicting 5-year survival in chondrosarcoma, with an AUC of 0.87 in the internal validation dataset. Although our DeepSurv model slightly outperformed the SORG algorithm in predicting 5-year survival (AUC of DeepSurv: 0.895), what makes our study more significant is that the influence of time on events is considered. Unlike SORG, which can only predict the binary outcome of 5-year survival, the DeepSurv model is more flexible and able to directly predict the patient’s survival function, thereby obtaining the probability of survival at any point in time. In addition, the neural network embedded in the DeepSurv model has great potential to learn from high-dimensional data and can be further enhanced by fitting images and genetic data, or by using multimodal information fusion techniques.

There are several limitations to consider in our study. Firstly, with the removal of one-third of the data used for internal validation, only 2,203 pieces of data were used for model training. Since chondrosarcoma tumors are mostly early-stage tumors (distant metastasis occurred in 128 of the 2203 patients), deep learning may not fully learn the characteristics of patients with advanced tumors. The prediction error curve also shows that the prediction performance of the DeepSurv model is significantly better than that of other models for patients with longer survival (**Figures 4**, **5**). Secondly, since the data are from national databases, some known prognostic factors [such as pathologic fracture (6) and biomarkers (2)] were not available. Thirdly, the model in this study has not been externally validated. Although we have adopted measures such as data segmentation and cross-validation in model development, the generalization and reliability of the model need to be further validated using other data sets. Fourthly, personalized treatment recommendations are another advantage of the DeepSurv algorithm (12, 18) but were not validated in this study because of the lack of treatment data. Due to the linear fitting of variables by the classical Cox model, the model recommended a constant treatment plan for all patients according to the calculated hazard ratio (HR) value. However, DeepSurv can make personalized treatment recommendations for different patients based on the complex non-linear relationship between the variables fitted by the model (12), which is more in line with real-world rules. For example, the use of chemotherapy in patients with chondrosarcoma is still controversial (1). By fitting the complex factors that affect the efficacy of chemotherapy, a treatment recommendation system based on deep learning may suggest the appropriate treatment for each individual.

To conclude, this study evaluated and compared the performance of two deep learning-based algorithms and two conventional methods for predicting overall survival in patients with chondrosarcoma. Overall, deep learning algorithms showed excellent discriminating capabilities, calibration, and stability in survival prediction. DeepSurv performed best in terms of discrimination and model calibration and was incorporated into a web-based application for clinical use. Further extension of the models developed in this work—considering specific aspects such as prognostic biomarkers, and image data—is necessary for future studies in order to encourage their widespread use in orthopedic oncology clinics for customized treatment planning and monitoring.

## Data availability statement

Publicly available datasets were analyzed in this study. This data can be found here: https://seer.cancer.gov/.

## Ethics statement

Because the SEER database is a publicly available database of de-identified patient data, no ethics committee review was required for its use in this project.

## Author contributions

YW, JC, and LY contributed to the conception and design of the study. LY organized the database. LY and NG performed the statistical analysis. LY, FA, YK, and YZ wrote the first draft of the manuscript. All authors contributed to manuscript revision, read, and approved the submitted version. The first two authors contributed equally to this work. The last two authors contributed equally to this work.

## Funding

This study was supported by the National Key R&D Program of China (2020YFC2006004-05).

## Conflict of interest

The authors declare that the research was conducted in the absence of any commercial or financial relationships that could be construed as a potential conflict of interest.

## Publisher’s note

All claims expressed in this article are solely those of the authors and do not necessarily represent those of their affiliated organizations, or those of the publisher, the editors and the reviewers. Any product that may be evaluated in this article, or claim that may be made by its manufacturer, is not guaranteed or endorsed by the publisher.
