# Supplementary material for: Deep learning models for predicting the survival of patients with chondrosarcoma based on a surveillance, epidemiology, and end results analysis
Source: Front Oncol. 2022 Aug 22;12:967758. doi: 10.3389/fonc.2022.967758 (PMC9442032; doi:10.3389/fonc.2022.967758)
Supplement: Supplementary file 1 [file Table_1.docx]

Supplementary Material E1

Table S1. The original name of variables in the SEER database and the specific details of each categorical variable.

|  | **Original variable name** |
| --- | --- |
| **Year of diagnosis** | Year of diagnosis |
| *2004-2010* | 2004, 2005, 2006, 2007, 2008, 2009, 2010 |
| *2011-2015* | 2011, 2012, 2013, 2014, 2015 |
| **Age** | Age recode with single ages and 100+ |
| **Gender** | Sex |
| *Female* | Female |
| *Male* | Male |
| **Histological type** | ICD-O-3 Hist/behav |
| *Conventional* | Conventional |
| *Dedifferentiated* | Dedifferentiated |
| **Primary site** | Primary Site - labeled |
| *Extremity* | C40.0, C40.1, C40.2, C40.3, C40.8, C40.9, |
| *Axial skeleton* | C41.1, C41.2, C41.4 |
| *Other* | C41.8, C41.9, C41.3, C41.0 |
| **Stage** | Derived AJCC Stage Group, 6th ed (2004-2015) |
| *I* | IA, IB |
| *II* | IIA, IIB |
| *III* | III |
| *IV* | IVA, IVB, IVNOS |
| *Missing* | Unknown |
| **Grade** | Grade (thru 2017) |
| *Well differentiated* | Well differentiated; Grade I |
| *Moderately differentiated* | Moderately differentiated; Grade II |
| *Poorly differentiated* | Poorly differentiated; Grade III |
| *Undifferentiated* | Undifferentiated; anaplastic; Grade IV |
| *Missing* | Unknown |
| **Surgery** | RX Summ--Surg Prim Site (1998+) |
| *No* | 0 |
| *Local treatment* | 15, 19, 25, 26 |
| *Radical excision with limb salvage* | 30 |
| *Amputation* | 40, 41, 42, 50, 51, 52, 53, 54 |
| *Missing* | 90, 99 |
| **Radiotherapy** | RX Summ--Surg/Rad Seq |
| *No* | No radiation and/or cancer-directed surgery |
| *Yes* | Intraoperative rad with other rad before/after surgery,  Radiation after surgery,  Radiation prior to surgery |
| **Chemotherapy** | Chemotherapy recode (yes, no/unk) |
| *No* | No/Unknown |
| *Yes* | Yes |
| **Tumor size** | CS tumor size (2004-2015) |
| *Missing* | 989, 990, 991, 992, 993, 994, 995, 996, 997, 998, 999, Blank(s) |
| **Number of tumors** | Total number of in situ/malignant tumors for patient |
| *1* | 1 |
| *> 1* | 2, 3, 4 |
| **Tumor extension** | CS extension (2004-2015) |
| *No break in periosteum* | 100, 200 |
| *Extension beyond periosteum* | 600, 400, 300, 350, 310 |
| *Further extension* | 700, 800, 820, 850 |
| *Missing* | 999, Blanks |
| **Distant metastasis** | CS mets at dx (2004-2015) |
| *No* | 0 |
| *Yes* | 30, 35, 40, 50, 53, 60 |
| *Missing* | 999, Blanks |
| **Survival months** | Survival months |
| **Status** | Vital status recode (study cutoff used) |
| *Alive* | Alive |
| *Dead* | Dead |
